# Trends in Water Level and Flooding in Dhaka, Bangladesh and Their Impact on Mortality

**DOI:** 10.3390/ijerph120201196

**Published:** 2015-01-22

**Authors:** Insa Thiele-Eich, Katrin Burkart, Clemens Simmer

**Affiliations:** 1 Meteorological Institute, University Bonn, Auf dem Huegel 20, D-53121 Bonn, Germany; E-Mail: csimmer@uni-bonn.de; 2 Climatology Laboratory, Geography Institute, Humboldt-Universität zu Berlin, Unter den Linden 6, D-10099 Berlin, Germany; E-Mail: katrin.burkart@geo.hu-berlin.de

**Keywords:** water level, flooding, mortality, climate change, extreme value theory, Bangladesh

## Abstract

Climate change is expected to impact flooding in many highly populated coastal regions, including Dhaka (Bangladesh), which is currently among the fastest growing cities in the world. In the past, high mortality counts have been associated with extreme flood events. We first analyzed daily water levels of the past 100 years in order to detect potential shifts in extremes. A distributed lag non-linear model was then used to examine the connection between water levels and mortality. Results indicate that for the period of 2003–2007, which entails two major flood events in 2004 and 2007, high water levels do not lead to a significant increase in relative mortality, which indicates a good level of adaptation and capacity to cope with flooding. However, following low water levels, an increase in mortality could be found. As our trend analysis of past water levels shows that minimum water levels have decreased during the past 100 years, action should be taken to ensure that the exposed population is also well-adapted to drought.

## 1. Introduction

Climate change is expected to impact the hydrological cycle due to both increased temperatures, thereby leading to changes in snow and ice regimes, as well as through shifts in the precipitation distribution [1,2]. Especially shifts in hydrological extremes leading to floods and droughts can have devastating economic and social effects such as loss of land, crops, or livestock, an increase in diseases, or even death [3].

Under a changing climate, extreme floods may increase [4], aggravating the situation for millions of people [5], in particular in South Asian coastal cities. The OECD report [6] places Dhaka, the capital of Bangladesh, among the top five most vulnerable coastal cities. Dhaka is currently threatened by a range of natural hazards such as earth quakes, tropical cyclones and—on an almost annual basis—flooding [7,8]. The effects of some of these threats can be counter-acted in part by improved disaster risk reduction measures, but economic risk exposure is nevertheless expected to rise with the strongly growing assets in developing regions, in particular during the next two decades [9]. In addition, despite a comparatively low fertility rate of 2.2 children per woman, the population of Dhaka is anticipated to increase from roughly 12 million inhabitants in 2000 to 16.8 million in 2015 [10] and to even larger numbers by the end of the century due to urban migration and the large proportion of young adults expected to become parents within the next years. Due to economic growth and growing population alone, the people of Dhaka already experience an increase in risk due to flooding during the coming years. Any additional impact of climate change e.g., on flooding will further endanger the future of the citizens of Dhaka [11].

**Figure 1:**
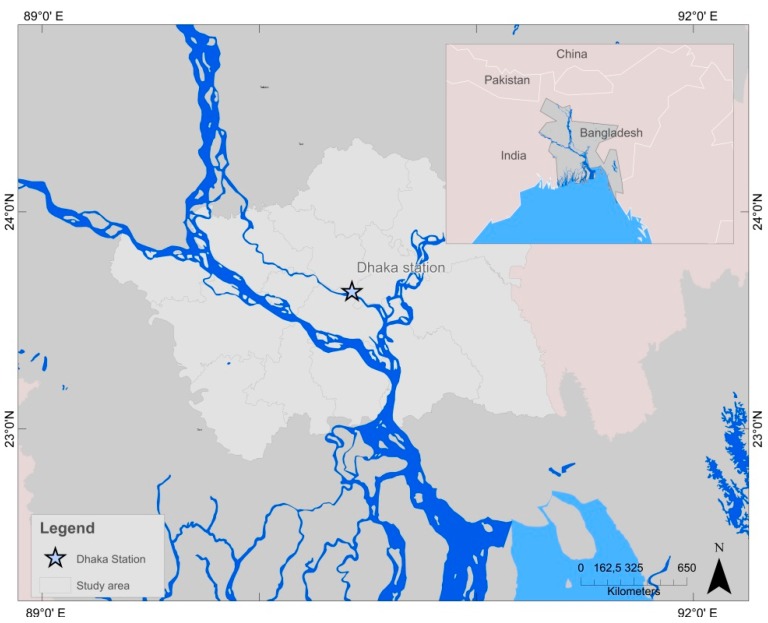
Map of study area, showing the river systems immediately surrounding Dhaka City as well as the location of the water level station.

Flooding in Dhaka is quantified by the exceedance of pre-determined danger levels at water level measurement stations operated by the Bangladesh Water Development Board. Flooding in the megacity is largely impacted by the close proximity to the confluence of the Ganges and Brahmaputra rivers upstream, as well as the conjunction with the Meghna river further downstream (Figure 1). The risk of flooding is aggravated through rapid urbanization and concurrent encroachment on retention areas, as well as increasing problems with both the natural and man-made drainage system. Particularly devastating flood years include the recent flooding of 2007, as well as the flood of 2004, during which over 30 million people were homeless in Bangladesh, with over 40% of Dhaka inundated. Water logging and drainage congestion enhance river flooding and its detrimental effect, which led the government of Bangladesh to ban the use of polyethylene shopping bags in 2002 [12]. Many of Dhaka’s residents, in particular slum dwellers, inhabit flood-prone areas, and only the western part of the city is currently protected by an embankment. Thus, inundation usually affects the poor more strongly, creating a social inequity [13]. Risks of flooding include both short- and long-term health risks such as e.g., gastrointestinal diseases, an increase in vector-borne diseases, psychological effects and possibly death [14,15]. For example, both the flood of 2004 and 2007 led to faecal contamination of drinking water sources in Dhaka due to drainage congestion problems [16,17]. Because the three most devastating floods occurred within the past 25 years, a general increase in extreme events is perceived [11,18,19].

Low water levels are also coming into focus as the groundwater level in Dhaka has receded in past years, with low river levels further depleting the available water resources. Aside from hardships for the day-to-day life, droughts also lead to health-related risks. Dey *et al.* [20] found that in Northwestern Bangladesh, droughts lead to increased levels of gastrointestinal diseases as well as dysentery compared to normal years.

Aside from the negative effects on health, both floods and droughts impact the mortality of the exposed population, as substantiated already for floods in Asian countries by Jonkman *et al.* [21]. Hashimoto *et al.* [22] used a numerical 2D flood simulation model to estimate flood-related health risks for Dhaka, and found a connection between maximum inundation depth and mortality. In a review of 35 epidemiological studies, Alderman [14] found that mortality can increase up to 50% in the first year after an extreme flood. Heavy flooding is assumed to increase mortality both through direct and indirect effects [23,24]. Direct effects include accidents with power lines and drowning, which are concurrent with the flooding (*i.e.*, do not exhibit a time lag). Indirect effects however, can lead to a higher morbidity due to e.g., gastrointestinal diseases [16], and could increase mortality with a time lag.

Because of these links between environmental factors and mortality, climate change is expected to impact mortality [25]. To understand the behavior of flooding and its links to mortality in the future, we assessed the past and current situation in Dhaka. We hypothesize that water levels have changed in frequency, magnitude and duration during the past century, and that an increase in mortality can be in parts related to extreme water levels. We first present an overview on observed water levels during the past 100 years in Dhaka City and then approach statistically possible links to mortality. In particular, we focus on rare—but particularly high-risk—events using extreme-value theory.

## 2. Data and Methodology

### 2.1. Data

#### 2.1.1. Hydrometeorological Data

Daily water levels for Dhaka covering the time period 1909–2009 were obtained from the Bangladesh Water Development Board. Both low and high tide water level are recorded on a daily basis and used for the analysis of annual minima and maxima, respectively. The data set originally has a total of 11.5% of missing values, which were imputed using the water level station Mirpur adjacent to the station in Dhaka. The missing values were estimated based on a combination of the mean annual cycle of Dhaka station and a linear regression between the residuals at both stations. The model fit is then used to predict the residuals for the missing days at Dhaka from residuals at Mirpur. This was validated both by testing that the mean and variance of the original and imputed data set are equal (two sample *t*-test, *p* = 0.15; *F*-test, *p* = 0.14), as well as by using residuals at Mirpur to predict the entire time series of Dhaka in a *k*-fold cross validation (RMSE = 0.20 m). The low RMSE value also shows that the method is suitable when imputing missing values to create a longer data set with fewer gaps.

The Bangladesh Water Development Board defines a water level of 6 m or higher as having crossed the danger level. For the time period 1909–2009, average number of days above danger level per year is 5, while in 2003–2007 the number of days above danger level was 18 and 1 in 2004 and 2007, respectively.

Three hourly values of temperature measured at the airport station in Dhaka were provided by the Bangladesh Meteorological Department from which daily mean values were calculated when the measurements were complete for a given day. Gaps in the time series of daily mean temperature due to missing measurements were replaced by linear interpolation. Lagged temperature variables were calculated as averages of the actual and the previous day (averages of lag 0–1) as well as the actual and the recent 13 days (averages of lag 0–13).

#### 2.1.2. Mortality Data

The Sample Vital Registration System (SVRS)—a core activity of the Bangladesh Bureau of Statistics (BBS)—collects vital information, such as birth and death events, of a sample population of approximately one million people. The SVRS collects data under a dual recording system: Events are recorded by a local registrar when they occur, and they are also registered retrospectively by officials from the Upazila division of the BBS on a quarterly basis. Subsequently both data sets are compared by quality control personnel of the BBS. Only partially matched and non-matched events are subject to further verification through field visits. To any fatality a cause of death is attributed; however, the cause is not medically certified [26]. For the purpose of this study, maternity-related deaths were excluded, leading to in total 5770 death counts in the study population from 2003 to 2007. Table 1 provides an overview over the range, mean and standard deviation of daily death counts. For further information on the SVRS we refer to Burkart *et al.* [27,28].

**Table 1:** Descriptive statistics for hydrometeorological and mortality data. Trend analysis of past water levels is performed using both low and high tide measurements for 1909–2009, while the remaining columns describe the water level, temperature and mortality data used to study a possible increase in fatalities following extremely low and high water levels.

Statistic	Water Level (m)				Temperature (°C)	Mortality ^a^ (Count)
	1909–2009		2003–2007		2003–2007	2003–2007
	Low Tide	High Tide	Low Tide	High Tide		
Min	0.24	0.55	0.60	0.98	11.9	1
Mean	2.77	3.04	2.71	3.06	26.0	3.7
Max	7.55	7.58	6.65	6.68	32.6	12
Stdev	1.66	1.48	1.52	1.35	4.1	2.0

^a^ Information refers to daily death counts.

### 2.2. Methodology

#### 2.2.1. Extreme Value Theory

We apply the generalized extreme value (GEV) family of distributions to model the distribution of annual maximum water levels, assuming that the elements of the time series are independent and identically distributed and that they have a common distribution function F [29]. Distributions of the GEV family are described by the location parameter μ, the scale parameter σ, and the shape parameter ξ, with distribution functions of the form:

(1) $$G\left(z\right)=\exp\left\{-{\left[1+\text{ξ}\left(\frac{z-\text{μ}}{\text{σ}}\right)\right]}^{-\frac{1}{\text{ξ}}}\right\}$$

defined on $\left\{z:1+\frac{\text{ξ}\left(z-\text{μ}\right)}{\text{σ}}>0\right\}$, where −∞<μ<∞, σ>0 and −∞<ξ<∞. To estimate the parameters of the GEV distribution $\left(\tilde{\mu },\text{σ},\text{ ξ}\right)$ for annual minima, the water levels $z_{1},\ldots ,z_{m}$ need to be converted to $-z_{1},\ldots ,-z_{m}$, with $\tilde{\text{μ}}=\;-\text{μ}$. Statistics of the distribution such as the mean can then be computed by:

(2) $$E\left[z\right]=\{\begin{array}{c} \mu +\sigma \frac{\Gamma \left(1-\text{ξ}\right)-1}{\text{ξ}}\;\mathrm{if}\;\xi \neq 0,\;\xi <1,\\ \;\mu +\sigma \gamma \;\mathrm{if}\;\xi =0,\;\mathrm{and}\\ \infty \;\mathrm{if}\;\xi >0 \end{array}$$

where γ is the Euler-Mascheroni constant. For a return period $\frac{1}{p},\;$ the return level $z_{p}$ and the associated return level plot can be obtained from inverting Equation (1) and setting $y_{p}=\;-log(1-p)$:

(3) $$z_{p}=\;\begin{array}{c} \text{μ}-\frac{\text{σ}}{\text{ξ}}\left[1-y_{p}^{-\text{ξ}}\right],\;for\;\text{ξ}\neq 0,\\ \text{μ}-\text{σ}\;log\;y_{p\;},\;for\;\text{ξ}=0. \end{array}$$

Estimates were performed using the R package “ismev” [30].

#### 2.2.2. Distributed Lag Non-Linear Models

In order to analyze the relationship between water level and mortality, we employed Distributed Lag Non-linear Models (DLNM) using the R package “dlnm” [31]. DLNMs help to estimate temporal displacements and thus potential harvesting effects. The methodology requires a “cross-basis”—a two-dimensional space of functions that describes simultaneously the shape of the relationship along both the space of the water level and the lag dimension of its occurrence [32]. Relationships are estimated using smooth non-linear functions for both dimensions, the water level effect and the lag effect [32]. The cross-basis function was included into a generalized additive model (GAM). In order to remove long-term fluctuations in mortality, the models were adjusted for trend by including a counter variable for each day of the time series and fitting a penalized spline. Additionally, a categorical dummy variable for seasonal adjustment was incorporated to remove the mid- to long-term seasonal cycles in the series, as we aimed to investigate rather short- to mid-term influences. Smoothing parameters were chosen to minimize Un-Biased Risk Estimator for the models. Since temperature exerts a profound effect on mortality [28,33], we included a lagged temperature variable into the models. We tested a 2 days lag as well as a 2 weeks lag to account for more short-term heat effects as well as more delayed cold effects (Section 2.1.1). Results were mostly unaffected by the choice of temperature lag.

The basis for water level in the DLNM was centered at the median (2.7 m), which represents the reference point for the predicted effects. The DLNM requires predefined degrees of freedom (DF) for the water level and lag relationships. We tested DFs between 2 and 7 as a sensitivity analysis. The general conclusions were mostly unaffected by the number of DFs and for the final analysis we chose 4 DFs for water level and 3 DFs for lag days.

## 3. Results

The assessment of historic changes in water levels in Dhaka is followed by a closer look at the connection between mortality and extreme events.

### 3.1. Assessment of Historic Changes in Water Levels in Dhaka

Changes in frequency, magnitude and duration of flooding in Dhaka City during 1909–2009 are presented and possible trends discussed.

#### 3.1.1. Changes in Frequency and Magnitude

**Figure 2:**
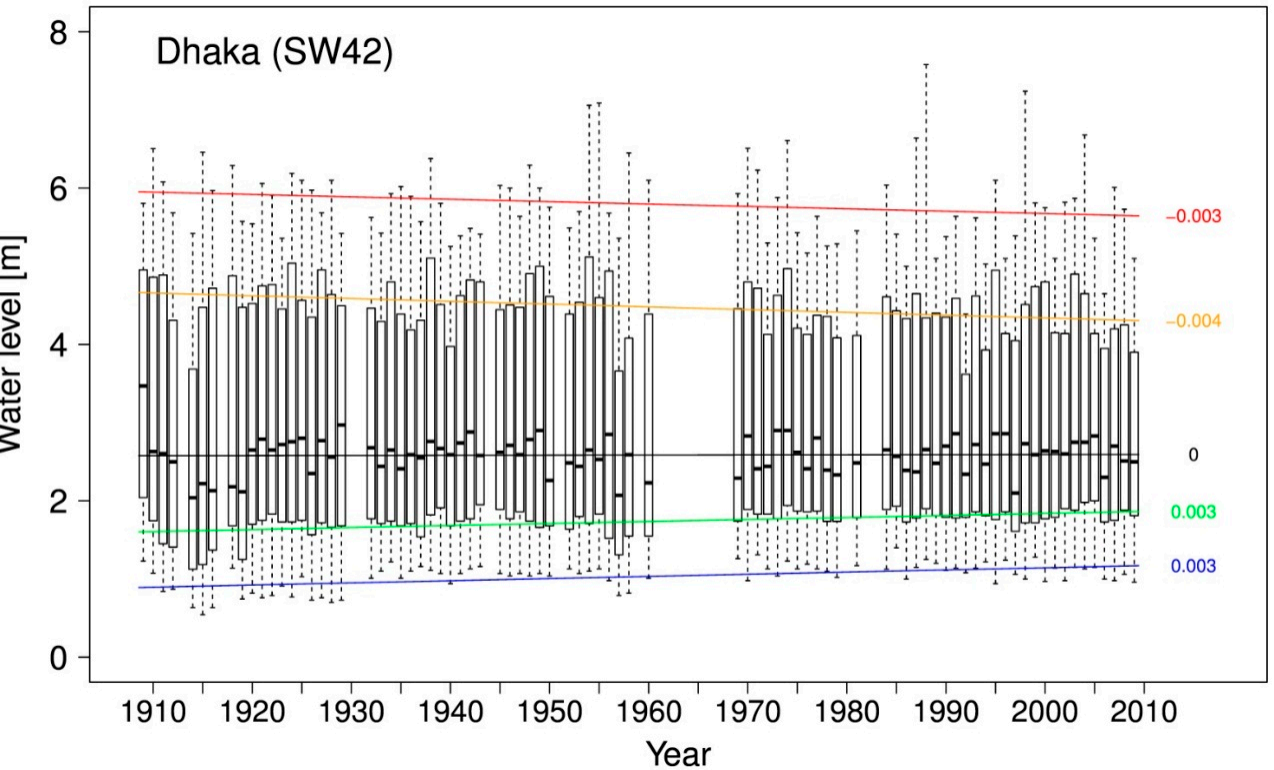
Annual boxplots for the water level station at Dhaka, 1909–2009. Regression lines are drawn through the upper and lower extremes (red and blue), as well as the 1st, 2nd, and 3rd quartile (green, black and orange, respectively). The slopes of the regression lines are printed on the right (m/year).

A quantile regression was performed on the high tide daily water level time series to see how the time series changes over time with respect to different quantiles such as the 1st, 2nd or 3rd quartile. Figure 2 shows that the lower quantiles including the median remain the same or increase slightly, with the higher quantiles decreasing slightly over time.

**Figure 3:**
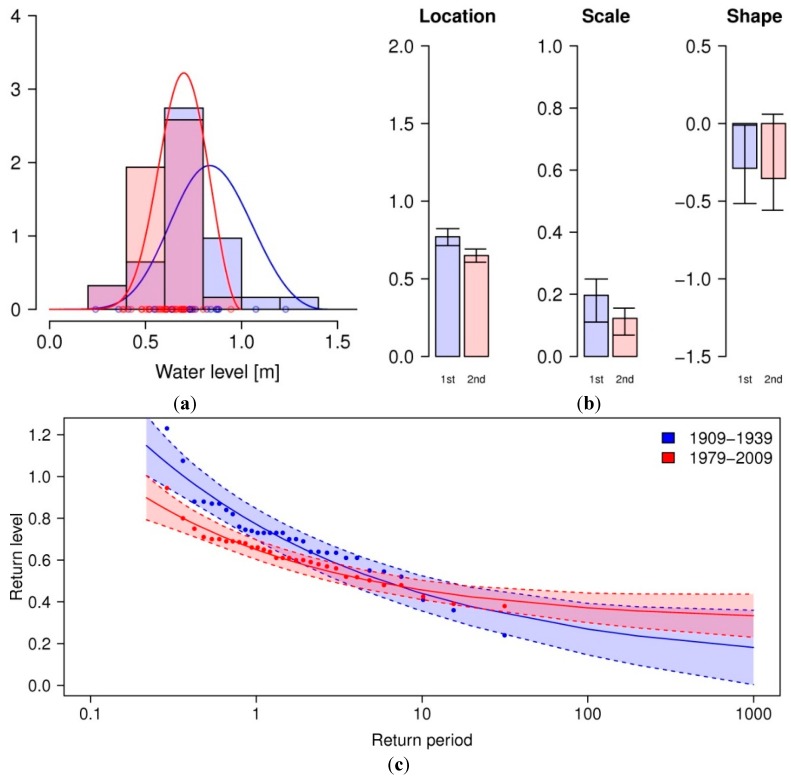
(**a**) shows the GEV distributions fitted to annual minimum water levels for 1909–1939 (blue) and 1979–2009 (red), estimated parameters location µ, scale σ and shape ξ are plotted in (**b**). Error bars depict 95% confidence intervals obtained from non-parametric bootstrapping (*R* = 500). Return level plots with 95% confidence intervals are shown in (**c**).

As seen from Figure 2, annual minimum water levels seem to slightly increase over the past century. To see if there is a trend in the variance of the maximum water level, *i.e.*, if the extremes—when they do occur- become more extreme over time, the parameters of a generalized extreme value distribution are estimated from the annual minimum water level for both 1909–1939 and 1979–2009. The shape parameter ξ is significantly below zero in both cases (at 0.05 for first period, 0.10 for second), reflecting the Weibull distribution applied to minimum time series. From the comparison of both distributions in Figure 3 it follows that contrary to the regression line above, the location parameter μ decreases at the 0.05 significance level, corresponding to a decrease in magnitude for the expected annual minima from 0.71 m to 0.61 m, while the scale parameter σ does not change. From the return level plot, it follows that all return periods of minimum water levels below the average annual minimum (return period = 1) have significantly lower return levels during the second time period than compared to the first, thereby a higher chance of occurring in a given year. The water level with a 1-year return period in the first period now has a return period of 0.5 years. For water levels above a return period of 1, no significant change can be found, which indicates that the extreme minimum water levels do not increase in frequency or magnitude.

**Figure 4:**
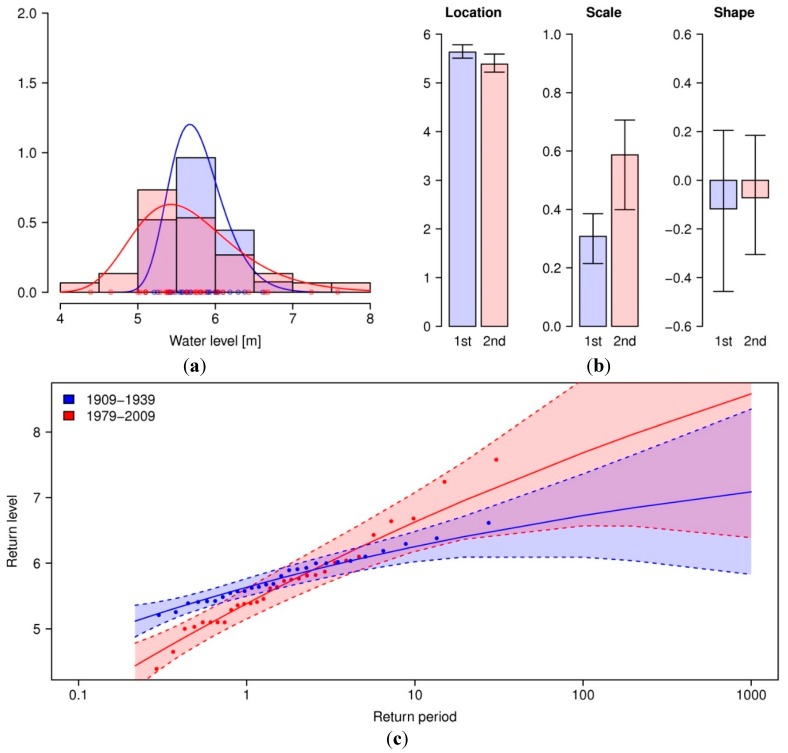
(**a**) shows the GEV distributions fitted to annual maximum water levels for 1909–1939 (blue) and 1979–2009 (red), estimated parameters location µ, scale σ and shape ξ are plotted in (**b**). Error bars depict 95% confidence intervals obtained from non-parametric bootstrapping (*R* = 500). Return level plots with 95% confidence intervals are shown in (**c**).

As seen from Figure 2, the trend in annual maximum water levels seems to be slightly negative. This contradicts current observations of extreme events having become more extreme. To see if there is a trend in the variance of the maximum water level, *i.e.*, if the extremes—when they do occur- become more extreme over time, the parameters of a generalized extreme value distribution are estimated from the annual maximum water level for both 1909–1939 and 1979–2009. Since ξ is not significantly different from zero in both cases, both can be considered Gumbel distributions. A deviance test [29] reveals a significant change over time in the Gumbel distribution once a step-wise trend is introduced for μ and σ as a covariate (D = 14.92, df = 2, *p* < 0.001). From the comparison of both distributions in Figure 4 it follows that while indeed the location parameter μ decreases at the 0.10 significance level, corresponding to the decrease in magnitude for the expected annual maxima from 5.78 m to 5.68 m, the scale parameter σ increases at the 0.05 significance level, which stands for a heavier tail or an increase in the more extreme events. From the return level plot, it follows that annual maximum water levels above 5.9 m have a lower return period during the second time period than compared to the first, thereby having a higher chance of occurring in a given year. For example, while a water level of 6.25 m has a return period of 10 years during the first time period, the return period decreases to 4.8 years during 1979–2009. The water level with a 10-year return period is now 6% higher at 6.63 m. This confirms that indeed the decrease of magnitude in annual maximum water levels does not contradict the publicly perceived increase in the most extreme events.

#### 3.1.2. Changes in Flood Duration

**Figure 5:**
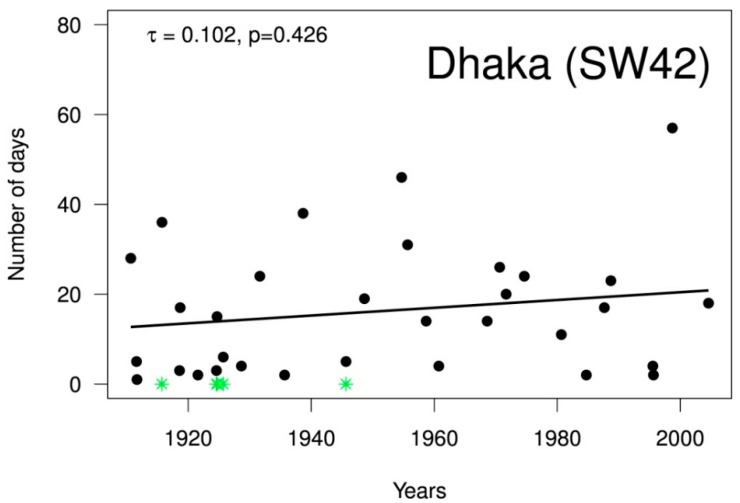
Duration of flooding Dhaka, 1909–2009. Years in which two flood events were counted as one are marked by green stars.

In addition to studying past trends in frequency and magnitude, the duration of flooding above danger level is also of interest. Figure 5 shows the duration of flood events, defined as consecutive days at or above the danger level registered for the water level station Dhaka. If two flood events are separated by only six days recording below danger level, they are considered as one continuous event (marked by green stars in Figure). For Dhaka, 32 events were counted between 1909–2009, with an average duration of 16.3 days. While no significant trend in flood duration was found for the past 100 years (Mann-Kendall trend test, *p* = 0.300), a slight decrease of 1.2 days/10 years is evident for the second half of the century (1953–2009, *p* = 0.117, figure not shown).

Overall, our results showed that while the magnitude and duration of average flood events in Dhaka have decreased or remained the same over time, the frequency of the most extreme events has increased. On the other side of the spectrum, minimum water levels have decreased over time.

### 3.2. Mortality and Extreme Events

Here, individual extreme hydrological events are studied to see if an impact on mortality can be detected. During the time period of available mortality data (2003–2007), two major flood events occurred in 2004 and 2007. Extreme drought events, which would be registered for water levels around 0.55 m (Figure 3), were not included in this time frame.

#### 3.2.1. Flood Event of 2004

**Figure 6:**
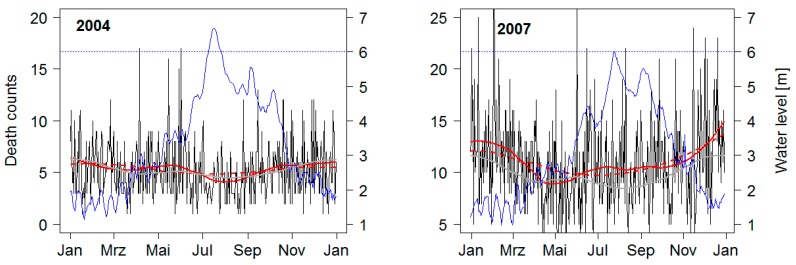
Time series plots of water level in Dhaka (**blue**) and daily deaths in flooded zilas in 2004 and 2007 (**black**). Red solid line displays loess smoothed values using an α of 0.1, the dashed red lines displays loess smoothed values using an α of 0.25. The grey line represents the average mortality from 2003 to 2007.

Heavy rainfalls during the beginning of July over large parts of the Ganges, Brahmaputra and Meghna catchments, accumulating over 300 mm in less than 7 days, resulted in heavy flooding throughout up to 50% of the country. Figure 6 demonstrates the elevated water levels in Dhaka surpassing the danger level of 6 m during most of July, with up to 40% of the capital being inundated. In September, a second flood peak can be seen due to a localized monsoon depression with heavy precipitation, bringing the total mortality count to 730 [18]. The mortality distribution shows the typical seasonal pattern with the highest levels occurring during winter, the lowest during the monsoon, and a secondary maximum during the pre-monsoon (although variations are less pronounced than during other years). During and after the flood event of 2004, mortality shows a clear trough with rather little noise in the daily mortality data.

#### 3.2.2. Flood Event of 2007

In 2007, excess rainfall in the Brahmaputra and Meghna catchments resulted in the first pronounced water level peak in the middle of July, with a second peak following and at the beginning of September. A total of 49 Zilas were affected with 55% of all roads flooded. While the water level station located in Dhaka did not cross the BWDB prescribed danger level of 6 m, the greater Dhaka area was considered as heavily flooded, with a total mortality count of 649 attributed to flooding [34]. Generally, seasonal variations in mortality are more pronounced in 2007 compared to other years and the annual mortality level is above average. During the monsoon, mortality is elevated and does not decline as usually after the secondary premonsoon peak. Several short-term peaks in daily mortality counts can be observed. However, these peaks do not exceed the general noise level.

### 3.3. Relationship between Water Level and Mortality in Greater Dhaka

**Figure 7:**
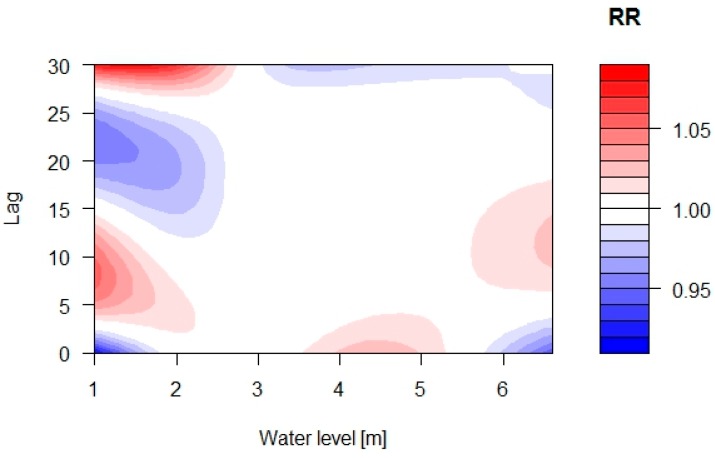
Contour plot of the relative risk (RR) of all-cause mortality along water level and lags, with reference to the median water level using a distributed lag non-linear model.

Contour plots demonstrate variations in RR at low and high water levels, whilst in between (at water levels of approximately 2.5 to 5.5 m), few variations in RR can be observed (Figure 7). At lower water levels (between water levels of 1 and 2.5 m) there is a varying pattern with increasing as well as decreasing mortality over the 30 days period. At a high water level of above 6 m, we observed a small decrease in mortality at lags of 0 to 3 days followed by an increase in mortality between lags of 6 and 17 days. However, few of the effects depicted in the contour plots were significant.

**Figure 8:**
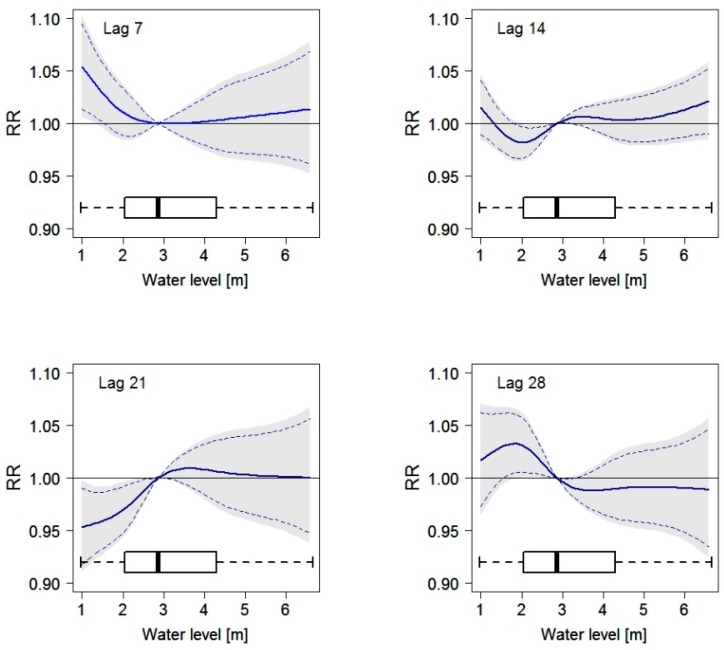
Relationship between the daily number of deaths and the water level in Dhaka for a lag period of 7, 14, 21 and 28 days using a distributed lag non-linear model. Curves are adjusted for trend, season, and day of the month. Grey areas display upper and lower 95% confidence intervals; dashed lines display upper and lower 90% confidence intervals. Boxplots for the water level distribution are included at the bottom of each plot.

In Figure 8, the association between water level and mortality is displayed for selected lag periods (mostly significant lag periods were included in this figure). At lag 7, there is a significant increase in mortality with decreasing water level below 3 m, highlighting an adverse effect of low water levels (or drought). With growing lag period this “drought effect” diminishes and at lag 21 days there is a significant increase in mortality with increasing water levels up to approximately 3 m. At a lag of 28 days, the relationship between water level and mortality changes again and mortality increases with decreasing water level, *i.e.*, a drought effect. However, this finding is only significant on a 90% level. For a lag period of 7 and 14 days we observed a minimal increase in mortality with increasing water level above the median. This increase is, however, not significant.

**Figure 9:**
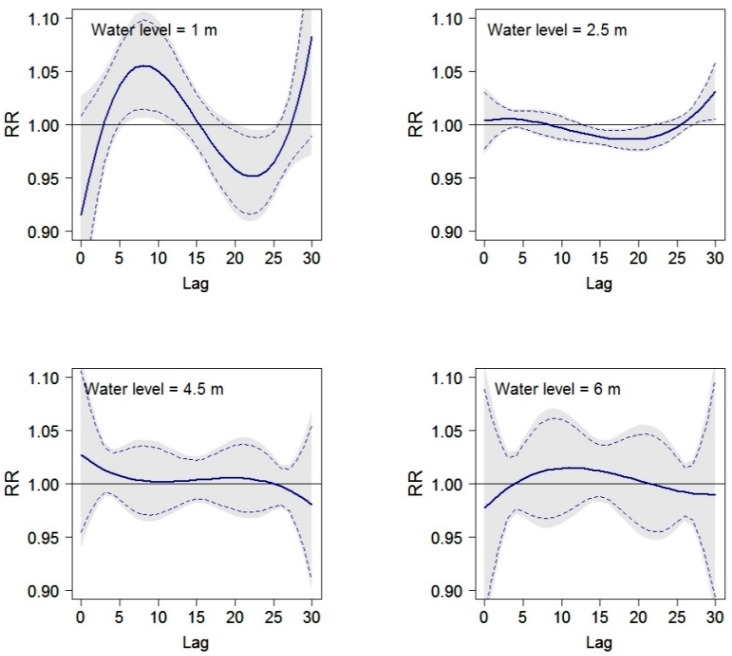
Plots of the relative risk of all-cause mortality at water levels of 1 m, 2.5 m, 4.5 m and 6 m along lags, with reference at the median water level using distributed lag non-linear models. Outputs are adjusted for trend, season, and day of the week. Grey areas display upper and lower 95% confidence intervals; dashed lines display 90% upper and lower 95% confidence intervals.

In Figure 9, the relative risk of all-cause mortality along lags is displayed for selected water levels. Again, these outputs underline the relevance of low levels and their effect on mortality. At a water level of 1 m we observed an increase in mortality up to a lag of 7 days. After 7 days, mortality decreases and reaches its lowest point at a lag of approximately 23 days before it starts rising again. At water level 2.5 m, mortality decreases slowly but significantly up to a lag of 21 days after which it starts rising again.

## 4. Discussion

We hypothesized that water levels have changed in frequency, magnitude and duration during the past century, and that mortality increases with or following extreme water levels. Our analysis suggests that water levels have indeed changed over the course of the past century. While the magnitude and duration of average flood events decreased, the frequency of extreme flood events has increased. Low water levels have also changed, with a significant decrease in the annual minimum water level most noticeable when we compare the time periods 1909–1939 and 1979–2009. A rise in mortality following extreme flood events could not be substantiated, but an increase in relative risk of death was found as water levels decrease.

Our results concerning trends in flooding seem to be at variance with the results from Mirza *et al.* [35], who did not find any consistent changes in peak discharges for the Ganges, Brahmaputra and Meghna using mean-based statistics. Since the discharges of this three-river system should be related, although not directly, to changes in Dhaka’s water levels, this discrepancy requires some thought. Concurring with Mirza *et al.* [35], we did not find an increase in mean extreme event indicators. The average annual maximum water level even slightly decreased (*p* < 0.10). However, the estimated GEV distribution for the second half of the century shows a more frequent occurrence of extreme events, suggesting that there is in fact a change in the variance of extreme events: when extreme events do occur, they become more extreme. Such a tendency cannot easily be detected from quantile regression or test statistics based on the data mean as applied by Mirza *et al.* [35].

While extreme events were seen to increase in magnitude and frequency, flood duration did not show any significant change. This is an important and somewhat positive finding as Aßheuer *et al.* [36] found a significant link between the number of days with high water levels and its negative impact on households located in informal settlements. An increase in damages due to flooding is therefore not likely due to an increase in flood duration, but perhaps more due to an increased exposure of the population and their assets [9].

Trends in low water levels have previously not been studied for Dhaka. Alam and Rabbani [11] found no change in annual average precipitation in daily rainfall data for Dhaka from 1971 to 2005, while the number of days without precipitation did increase. Since a positive relation between days without precipitation and low water levels seems physical reasonable, their results concur with our results that indicate the annual minimum water level has decreased over time.

We assumed that flooding could result in direct deaths through e.g. drowning, or could have indirect effects leading to an overload or breakdown of infrastructures, ultimately resulting in an increase of death rates. The spread of pathogens due to stagnating bodies of water and the increase of water-borne diseases, as well as a reduced access to health care and medical facilities, could contribute to a higher number of deaths. Milojevic *et al.* [37] reported a significant impact of flooding on respiratory diseases for up to 6 months after a flood event, but did not see any effects on gastrointestinal diseases or mortality when studying health effects in rural Bangladesh. Our results also show that high water levels have no or only a weak relationship with mortality for the urban area of Dhaka.

Death counts in the two recent flood events of 2004 and 2007 are lower compared to those of the previous three extreme flood events (3680 in 1987, 2379 in 1988, and 1050 in 1998 [18]). This absence of high water level effects on mortality could indicate an adequate adaptation to such events as discussed in Aßheuer *et al.* [38]. This is in line with Ashley and Ashley [39], who studied flood fatalities in the United States and found that behavior during floods was a risk factor for mortality. Flood policy makers are recommended to target specific groups and educate about the dangers of floods. With the much more frequent exposure of the Bangladeshi population to this natural hazard, the decline in mortality in recent years could in fact be due to a more educated and adapted community.

Less focus has been placed on the implications of droughts on mortality, in particular for an urban area. This study highlights a possible negative effect of low water levels and drought on mortality in Bangladesh. Explanations for this might be stagnating bodies of water or a general lack of dilution of freshwater and sewage systems, leading to contamination and the spread of pathogens [40,41]. In studies conducted in Bengal, the first outbreak of cholera in the year was associated with low precipitation and low river discharge. High precipitation and peak streamflow of rivers during the monsoon was associated with the second peak during the monsoon [42,43].

Studying the hydrological effects, in particular those of low water levels, on mortality in this urban setting is of utmost importance to assess the implications of climate change on the growing urban population in greater Dhaka. In this respect, we consider our approach as highly beneficial to assess the complex association between effect and response, and to better understand temporal displacement of mortality and harvesting. To our knowledge, there is no other study using extreme value theory, which proved more suitable to detect trends in extremes than quantile regression or test statistics based on the data mean, and the DLNMs, which are specifically designed to investigate the lag structure of environmental effects.

While the hydrological network of the Bangladesh Water Development Board provides extensive spatial coverage for the region, the daily water level time series is subject to several years of missing values which could not be imputed from neighboring stations for lack of data particularly for the years before 1953. Although the direct contribution of individual factors to past floods is difficult to measure, they certainly cause uncertainty in the assessment of trends in water level. Khalequzzam [19] names changes in riverbed characteristics such as siltation or external changes such as human interference with canals or the filling in of river channels to gain more accessible land for housing as possible factors in the creation of floods. Factors such as sea-level rise, subsidence, compaction of sediments in the enormous river delta, and a sinking groundwater table underneath Dhaka City all cause changes in land elevation, which are not taken into account in our trend analysis of historic water levels due to insufficient data. Possibly, satellite measurements could be used to give an indication of how strong elevation changes were in past years, but the coverage is not extensive enough to treat the data record of the past 100 years. The water level data could also be impacted directly as the Buriganga River contributes to recharging the Dupi Tila aquifer below Dhaka City [44].

A longer span of mortality data would also be beneficial, as high water level values may have an effect without these showing as significant, possibly due to low number of death counts in the greater Dhaka area and the overall survey sample size. Problems could also occur due to uncertainty in the registration of flood-related deaths and their exact location, as proposed by Milojevic *et al.* [45], who studied medium-term impact of flooding on mortality in the UK. Even using their extensive data set of mortality registrations for 1993–2006 linked to over 300 flood events, they found a counter-intuitive decrease in mortality the year after flooding, which could in part be due to uncertainty regarding the place of residence when registering death counts.

Furthermore, in a previous paper we analyzed seasonality of all-cause and cause-specific mortality [27]. Mortality was generally lower during summer and the rainy season and peaked during the cold season. With regard to diarrheal mortality, a secondary peak at the end of the rainy season could be observed in rural areas and areas with a low socioeconomic status, but variations were at the limit of detection and significance. Droughts or low water levels typically occur during the cold season when mortality is high. As it is difficult to separate the effects of water level and temperature on mortality, it is possible that we have not sufficiently adjusted for season. Our observed effect could then in part be due to cold temperature effects, but it is also possible that part of the cold effect found in Burkart *et al.* [28] is, in fact, a drought effect.

Our results nevertheless give an indication for several actions to assist the population of Dhaka in increasing their adaptive capacity [46,47] to natural hazards. While the Flood Forecast and Warning Center already provides accurate information and timely warnings regarding flood events, this network should be further developed to also include information regarding drought so the population, planning authorities and decision makers can adequately prepare. NGOs and international relief organizations should not focus mainly on the catastrophe management following extreme flood events and heat waves, but consider the impact of droughts and cold spells on mortality when preparing for natural disasters in the region.

Habiba *et al.* [48] studied drought adaptation measures of farmers in Northwestern Bangladesh and found that e.g., the provision of drought-tolerant crops or the establishment of community health care services would assist the community in coping with droughts. We propose extending these studies to include a focus on an urban setting, where the depleted groundwater table is an issue that can possibly exacerbate low river levels.

Structural as well as non-structural measures also need to be implemented to assist the population in coping with extreme events [47]. During the flood of 2004, 2 million city residents had limited access to drinking water as their supplies were contaminated, resulting in over 100,000 reported diarrhea cases in Dhaka alone [11,16]. This was due in large parts to submerged pumps operated by the Dhaka Water and Sewerage Authority. While the overall death count was low compared to previous flood events, it is nevertheless important that the pump and sleuce system is well-prepared to deal with an increase in extreme flood events, even though the average annual flooding may decrease. This holds particularly true since the population of Dhaka is expected to grow in future years, leaving more people affected by flooding. Despite this population growth, it is still important to ensure that flood plains and embankments are accessible and not filled up to create land. Additional measures could include the dredging of rivers [19].

Finally, a closer look at climate change and its impact on water levels in Dhaka is vital to adequately prepare the population for the expected changes until the end of the century. Using climate scenarios from four global climate models as input to the river modeling system MIKE11-GIS, Mirza [18] estimated that the mean flooded area is expected to increase by 29% for 0–2 °C of warming. While it is not yet feasible to derive urban water levels directly from global climate model or even regional climate model output, a closer look should be taken into downscaling methods connecting model output to urban water models such as MIKE URBAN to assess future impact of climate change on extreme water levels in Dhaka.

## 5. Conclusions

Our analysis suggests that water levels have indeed changed over the course of the past century. While the magnitude and duration of average flood events decreased, the frequency of extreme flood events has increased. Low water levels have also changed, with a significant decrease in the annual minimum water level most noticeable when we compare the time periods 1909–1939 and 1979–2009. A rise in mortality following extreme flood events could not be substantiated, but an increase in relative risk of death was found as water levels decrease.

While the current adaptation capacity of the population seems to be sufficient in dealing with mortality resulting from extreme flood events, action should be taken to ensure that these measures are continuously improved and sufficient to handle future changes in water levels. Further studies both on the public adaptive capacity as well as on the impact of climate change on water levels are therefore of importance, in particular with a focus on droughts in an urban setting.

## References

[1] Arnell N.W. (1999). Climate change and global water resources. Glob. Environ. Chang..

[2] IPCC (2013). Climate Change 2013: The Physical Science Basis. Contribution of Working Group I to the Fifth Assessment Report of the Intergovernmental Panel on Climate Change.

[3] IPCC (2014). Climate Change 2014: Impacts, Adaptation, and Vulnerability. Part A: Global and Sectoral Aspects. Contribution of Working Group II to the Fifth Assessment Report of the Intergovernmental Panel on Climate Change.

[4] Milly P.C.D., Wetherald R.T., Dunne K.A., Delworth T.L. (2002). Increasing risk of great floods in a changing climate. Nature.

[5] Few R. (2003). Flooding, vulnerability and coping strategies: Local responses to a global threat. Prog. Dev. Stud..

[6] Organization for Economic Cooperation and Development (OECD) (2007). Environment Working Paper. Screening Study: Ranking Port Cities with High Exposure and Vulnerability to Climate Extremes. Interim Analysis: Exposure Estimates.

[7] Risk and Poverty in a Changing Climate. http://www.preventionweb.net/files/9414_GARsummary.pdf.

[8] Revealing Risk, Redefining Development. http://www.preventionweb.net/english/hyogo/gar/2011/en/home/index.html.

[9] Patt A.G., Tadross M., Nussbaumer P., Asante K., Metzger M., Rafael J., Goujon A., Brundrit G. (2010). Estimating least-developed countries’ vulnerability to climate-related extreme events over the next 50 years. Proc. Natl. Acad. Sci. USA.

[10] United Nations (2006). World Urbanization Prospects: The 2005 Revision.

[11] Alam M., Rabbani M.D.G. (2007). Vulnerabilities and responses to climate change for Dhaka. Environ. Urban..

[12] (2006). Reazuddin and Team. Report: Banning Polyethylene Shopping Bags: A Step Forward to Promoting Environmentally Sustainable Development in Bangladesh.

[13] Brouwer R., Akter S., Brander L., Haque E. (2007). Socioeconomic vulnerability and adaptation to environmental risk: A case study of climate change and flooding in Bangladesh. Risk Anal..

[14] Alderman K., Turner L.R., Tong S. (2012). Floods and human health: A systematic review. Environ. Int..

[15] Lowe D., Ebi K.L., Forsberg B. (2013). Factors increasing vulnerability to health effects before, during and after floods. Int. J. Environ. Res. Public Health.

[16] Sirajul Islam M., Brooks A., Kabir M.S., Jahid I.K., Shafiqul Islam M., Goswami D., Nair G.B., Larson C., Yukiko W., Luby S. (2007). Faecal contamination of drinking water sources of Dhaka city during the 2004 flood in Bangladesh and use of disinfectants for water treatment. J. Appl. Microbiol..

[17] Islam A.S., Haque A., Bala S. (2010). Hydrologic characteristics of floods in Ganges–Brahmaputra–Meghna (GBM) delta. Nat. Hazards.

[18] Mirza M.M. (2011). Climate change, flooding in South Asia and implications. Reg. Environ. Chang..

[19] Khalequzzaman M.D. (1994). Recent floods in Bangladesh: Possible causes and solutions. Nat. Hazards.

[20] Dey N., Alam M., Sajjan A., Bhuiyan M., Ghose L., Ibaraki Y., Karim F. (2011). Assessing environmental and health impact of drought in the Northwest Bangladesh. J. Environ. Sci. Nat. Resour..

[21] Jonkman S.N. (2005). Global perspectives on loss of human life caused by floods. Nat. Hazards.

[22] Hashimoto M., Suetsugi T., Sunada K., ICRE (2011). Study on the flood simulation techniques for estimation of health risk in Dhaka city, Bangladesh. American Geophysical Union, Fall Meeting.

[23] Ahern M., Kovats R.S., Wilkinson P., Few R., Matthies F. (2005). Global health impacts of floods: Epidemiologic evidence. Epidemiol. Rev..

[24] Du W., FitzGerald G.J., Clark M., Hou X.Y. (2010). Health impacts of floods. Prehospital Disaster Med..

[25] Patz J.A., Campbell-Lendrum D., Holloway T., Foley J.A. (2005). Impact of regional climate change on human health. Nature.

[26] Bangladesh Bureau of Statistics (2008). Report on the Sample Vital Registration System 2007.

[27] Burkart K., Khan M., Kramer A., Breitner S., Schneider A., Endlicher W. (2011). Seasonal variations of all-cause and cause-specific mortality by age, gender, and socioeconomic condition in urban and rural areas of Bangladesh. Int. J. Equity Health.

[28] Burkart K., Schneider A., Breitner S., Khan M.H., Krämer A., Endlicher W. (2011). The effect of atmospheric thermal conditions and urban thermal pollution on all-cause and cardiovascular mortality in Bangladesh. Environ. Pollut..

[29] Coles S. (2001). An Introduction to Statistical Modeling of Extreme Values.

[30] ismev: An Introduction to Statistical Modeling of Extreme Values. R Package Version 1.35. http://CRAN.R-project.org/package=ismev.

[31] Gasparrini A. (2011). Distributed lag linear and non-linear models in R: The package dlnm. J. Stat. Softw..

[32] Gasparrini A., Armstrong B., Kenward M. (2010). Distributed lag nonlinear models. Stat. Med..

[33] Burkart K., Breitner S., Schneider A., Khan M.M., Krämer A., Endlicher W. (2014). An analysis of heat effects in different subpopulations of Bangladesh. Int. J. Biometeorol..

[34] Disaster Management Bureau: Ministry of Food and Disaster Management For Government of the Peoples Republic of Bangladesh with the assistance of comprehensive Disaster Management Programme (CDMP). http://www.ddm.gov.bd/.

[35] Mirza M.M.Q., Warrick R.A., Ericksen N.J., Kenny G.J. (2001). Are floods getting worse in the Ganges, Brahmaputra and Meghna basins?. Glob. Environ. Chang. Part B Environ. Hazards.

[36] Aßheuer T. (2014). Klimawandel und Resilienz in Bangladesch: Die Bewältigung von Überschwemmungen in den Slums von Dhaka.

[37] Milojevic A., Armstrong B., Hashizume M., McAllister K., Faruque A., Yunus M., Kim Streatfield P., Moji K., Wilkinson P. (2012). Health effects of flooding in rural Bangladesh. Epidemiology.

[38] Aßheuer T., Thiele-Eich I., Braun B. (2013). Coping with the impacts of severe flood events in Dhaka’s slums—The role of social capital. Erdkunde.

[39] Ashley S.T., Ashley W.S. (2008). Flood fatalities in the United States. J. Appl. Meteorol. Climatol..

[40] Hashizume M., Armstrong B., Hajat S., Wagatsuma Y., Faruque A.S., Hayashi T., Sack D.A. (2007). Association between climate variability and hospital visits for non-cholera diarrhoea in Bangladesh: Effects and vulnerable groups. Int. J. Epidemiol..

[41] Zhang Y., Bi P., Hiller J.E., Sun Y., Ryan P. (2007). Climate variations and bacillary dysentery in northern and southern cities of China. J. Infect..

[42] Akanda A.S., Jutla A.S., Islam S. (2009). Dual peak cholera transmission in Bengal Delta: A hydroclimatological explanation. Geophys. Res. Lett..

[43] Hashizume M., Wagatsuma Y., Faruque A., Hayashi T., Armstrong B. (2009). Climatic components of seasonal variation in Cholera incidence. Epidemiology.

[44] Morris B., Seddique A.A., Ahmed K.M. (2003). Response of the Dupi Tila aquifer to intensive pumping in Dhaka, Bangladesh. Hydrogeol. J..

[45] Milojevic A., Armstrong B., Kovats S., Butler B., Hayes E., Leonardi G., Murray V., Wilkinson P. (2011). Long-term effects of flooding on mortality in England and Wales, 1994–2005: Controlled interrupted time-series analysis. Environ. Health.

[46] Hess J., McDowell J., Luber G. (2012). Integrating climate change adaptation into public health practice: Using adaptive management to increase adaptive capacity and build resilience. Environ. Health Perspect..

[47] Faisal I.M., Kabir M.R., Nishat A. (2003). The disastrous flood of 1998 and long term mitigation strategies for Dhaka City. Nat. Hazards.

[48] Habiba U., Shaw R., Takeuchi Y. (2014). Farmers’ adaptive practices for drought risk reduction in the northwest region of Bangladesh. Nat. Hazards.

